# A Control Study on the Value of the Ultrasound Grayscale Ratio for the Differential Diagnosis of Thyroid Micropapillary Carcinoma and Micronodular Goiter in Two Medical Centers

**DOI:** 10.3389/fonc.2020.625238

**Published:** 2021-01-25

**Authors:** Zhijiang Han, Na Feng, Yidan Lu, Mingkui Li, Peiying Wei, Jincao Yao, Qiaodan Zhu, Zhikai Lei, Dong Xu

**Affiliations:** ^1^ Department of Radiology, Affiliated Hangzhou First People’s Hospital, Zhejiang University School of Medicine, Hangzhou, China; ^2^ Department of Ultrasound, The Cancer Hospital of the University of Chinese Academy of Sciences (Zhejiang Cancer Hospital), Institute of Basic Medicine and Cancer (IBMC), Chinese Academy of Sciences, Key Laboratory of Head & Neck Cancer Translational Research of Zhejiang Province, Zhejiang Provincial Research Center for Cancer Intelligent Diagnosis and Molecular Technology, Hangzhou, China; ^3^ Department of Ultrasonography, Zhejiang Xiaoshan Hospital, Hangzhou, China

**Keywords:** ultrasound gray scale ratio (UGSR), papillary thyroid microcarcinoma, micronodular goiter, diagnosis, echogenicity

## Abstract

**Objective:**

To investigate the value of ultrasound gray-scale ratio (UGSR) for the differential diagnosis of papillary thyroid microcarcinoma (PTMC) and micronodular goiter (MNG) in two medical centers.

**Methods:**

Ultrasound images of 881 PTMCs from 785 patients and 744 MNGs from 687 patients in center A were retrospectively analyzed and compared with 243 PTMCs from 203 patients and 251 MNGs from 198 patients in center B. All cases were confirmed by surgery and histology. The grayscale values of thyroid lesions and surrounding normal tissues were measured, and the UGSR was calculated. The optimal UGSR threshold for identifying PTMCs and MNGs in two medical centers was determined by receiver operating characteristic (ROC) curve, and the area under the curve (AUC), optimal UGSR threshold, sensitivity, specificity, positive predictive value, negative predictive value, and accuracy were compared between the two medical centers.

**Results:**

The UGSR values of PTMCs and MNGs in medical center A were 0.5537 (0.4699, 0.6515) and 0.8708 (0.7616, 1.0123) (Z = -27.691, *P* = 0), respectively, whereas those in medical center B were 0.5517 (0.4698, 0.6377) and 0.8539 (0.7366, 0.9929) (Z = -16.057, *P* = 0), respectively. The UGSR of PTMCs and MNGs did not differ significantly between the two medical centers (Z = -0.609, *P* = 0.543 and Z = -1.394, *P* = 0.163, respectively). The AUC, optimal UGSR threshold, sensitivity, specificity, positive predictive value, negative predictive value, and accuracy of the two medical centers were 0.898 vs. 0.918, 0.7214 vs. 0.6911, 0.881 vs. 0.868, 0.817 vs. 0.833, 0.851 vs. 0.834, 0.853 vs. 0.867, and 0.852 vs. 0.850, respectively.

**Conclusions:**

UGSR can quantify the echo intensity of PTMCs and MNGs and is therefore valuable for the differential diagnosis of the two diseases. The diagnostic efficacy was consistent between the two medical centers. This method should be widely promoted and applied.

## Introduction

In the ultrasound examination of thyroid nodules, the echogenicity of the thyroid gland and strap muscle is often used as a reference as observed by the physicians’ naked eyes, and it is divided into three to five levels. This method is called Thyroid Imaging Reporting and Data System (TIRADS). The Korean-TIRADS classifies lesions into three types according to echo intensity: hypoechoic, isoechoic, and hyperechoic. The American College of Radiology (ACR) TIRADS divides lesions into four levels: very hypoechoic, hypoechoic-isoechoic, hyperechoic, and anechoic. The European-TIRADS includes five levels: markedly hypoechoic, mildly hypoechoic, isoechoic, hyperechoic, and anechoic (1–3). Although hypoechoic in the Korean-TIRADS, very hypoechoic in the ACR-TIRADS, and markedly hypoechoic in the European-TIRADS cover different ranges, these levels are considered as suspicious malignant nodules. The diagnostic performance can also show differences. Even if the same TIRADS is used, the subjective assessment can lead to diagnostic differences (4), particularly in the thyroid gland, which differs in thickness from the strap muscle, and the echo intensity can vary between different sections of the same strap muscle. Assessment of the signal intensity in nodules and strap muscles is greatly influenced by subjective factors (5). In addition, as the echo intensity of the thyroid nodule changes from weak to strong, the grayscale value changes from low to high and the image from black to white; the grayscale value is a continuous variable, theoretically, and different variables contribute to the diagnostic efficacy (5). Although a rough grading of 3–5 has been recognized by many scholars (1–5), the gray-scale value of the thyroid or strap muscle is not necessarily the optimal threshold for distinguishing benign and malignant nodules. Quantification of the echo intensity would facilitate the differential diagnosis of benign and malignant thyroid nodules.

The ultrasound echo intensity can be affected by factors such as gain, dynamics, operator, and type of machine among others. The diagnostic value of echo intensity obtained by direct measurement is limited; however, under the premise of a standardized operation, the echo intensity of the nodule and surrounding thyroid tissues increases or decreases simultaneously despite changes in the factors affecting each; that is, the intensities designated as “low”, “equal,” and “high” maintain a balance. Therefore, the echo intensity of the nodule can be indirectly quantified by measuring the ultrasound gray-scale ratio (UGSR) of the nodule to the surrounding thyroid tissues, which is more objective than the naked eye assessment of levels 3–5. Including our previous studies, only four reports of the quantification of thyroid nodules using UGSR have been published to date (5–8). These reports all suggest that the UGSR of malignant nodules is lower than that of solid benign nodules but higher than that of cystic benign nodules. Although the diagnostic efficacy of UGSR is significantly higher than that of the traditional three to five level method, the four published studies have certain limitations, such as the inclusion of a single medical center and the use of the same ultrasound scanner in two studies. Confirming the diagnostic value of UGSR using different scanners in different medical centers would improve the accuracy of studies for clinical application.

In this study, UGSR values and their diagnostic performance were compared between two medical centers to determine the utility and consistency of UGSR for the differential diagnosis of PTMC and MNG and to provide a potential reference for improving TIRADS.

## Materials and Methods

### Patient Selection

**Figure 1:**
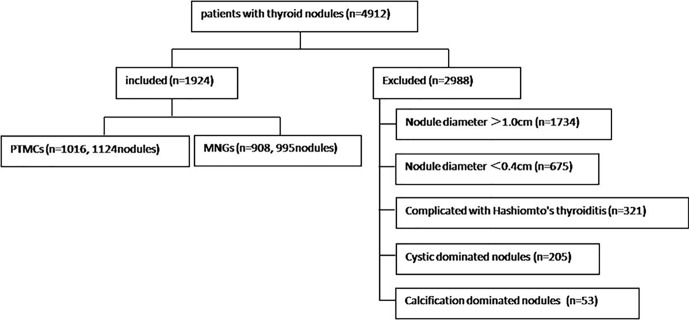
Flow chart of study participants.

The study was performed in accordance with the Helsinki Declaration ethics guidelines and approved by the ethics committees of the two institutions. The study included 3,712 consecutive cases of thyroid nodules treated in the Affiliated Hangzhou First People’s Hospital, Zhejiang University School of Medicine (center A for short) between June 2017 and June 2020 and 1,200 consecutive cases from The Cancer Hospital of the University of Chinese Academy of Sciences (Zhejiang Cancer Hospital) (center B for short) between June 2019 and June 2020. Thyroid nodules with a diameter 1.0 cm or <0.4 cm, cystic-dominated nodules (where the cystic component was >50% of the nodule volume) (9, 10), Hashimoto’s thyroiditis, and calcification-dominated nodules (unmeasurable due to obvious calcification) (5) were excluded. Finally, 1,472 cases and 1,625 thyroid nodules from center A and 452 cases and 494 thyroid nodules from center B that met the inclusion criteria were analyzed. There were 365 men [mean age, 52 (42–59) years] and 1,508 women [mean age, 51 (42–57) years]. **Figure 1** is a flow chart indicating the screening process and characteristics of the study participants.

### Ultrasound Examination

Five ultrasound scanners were used in center A as follows: MyLab 70 XVG (Genova, Italy), Esaote MyLab Classic C (Genova, Italy), Esaote Mylab 90 (Genova, Italy), Mindray (Shenzhen, China), and Hitachi (Tokyo, Japan). The scanners used 5–10 MHz broadband linear array probes with a central frequency of 7.5 MHz. Two ultrasound scanners were used in center B as follows: TOSHIBA Aplio 400 (Tochigi, Japan) and GE Logiq E9 (Wauwatosa, USA). The patient’s posture and content scanned were the same in the two medical centers. Patients were placed in the supine position with neck hyperextended; transverse, longitudinal and oblique sections were scanned, and the nodule data were recorded as follows: number, shape, size, calcification, internal echo, halo around the boundary, internal and peripheral blood flow, and bilateral cervical lymph nodes (5).

### Image Analysis

**Figure 2:**
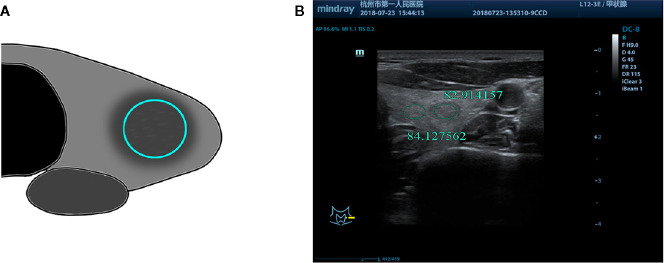
**(A)** Schematic diagram of gray-scale measurement of nodules with uniform echo intensity. **(B)** F, 53-years-old, MNG in the left thyroid lobe, nodules with uniform echo intensity, UGSR = 82.914157/84.127562 = 0.9856.

**Figure 3:**
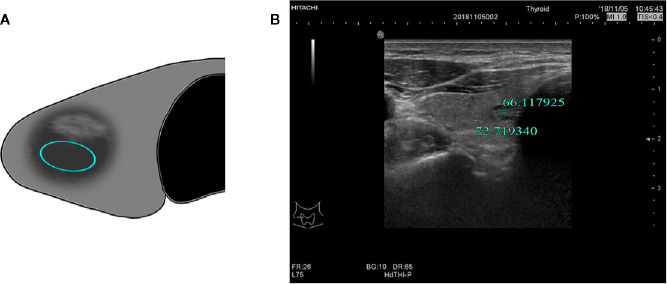
**(A)** Schematic diagram of the gray-scale measurement of a nodule with uneven echo intensity by a certain echo intensity. **(B)** M, 57-years-old, MNG in the right lobe of the thyroid with uneven nodule echo intensity, UGSR = 66.117925/72.719340 = 0.9092.

**Figure 4:**
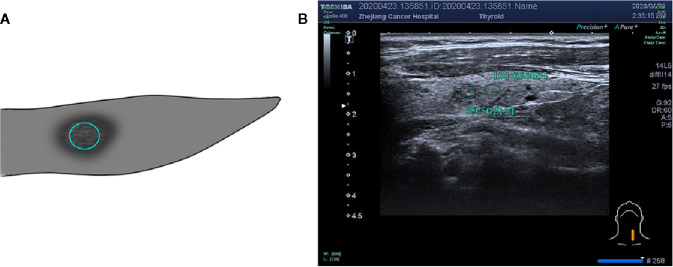
**(A)** Schematic diagram of gray-scale measurement of a nodule with uneven or not determined echo intensity. **(B)** F, 53-years-old, MNG in the left thyroid lobe, nodule with uneven echo intensity, UGSR = 79.502121/101.656061 = 0.7821.

**Figure 5:**
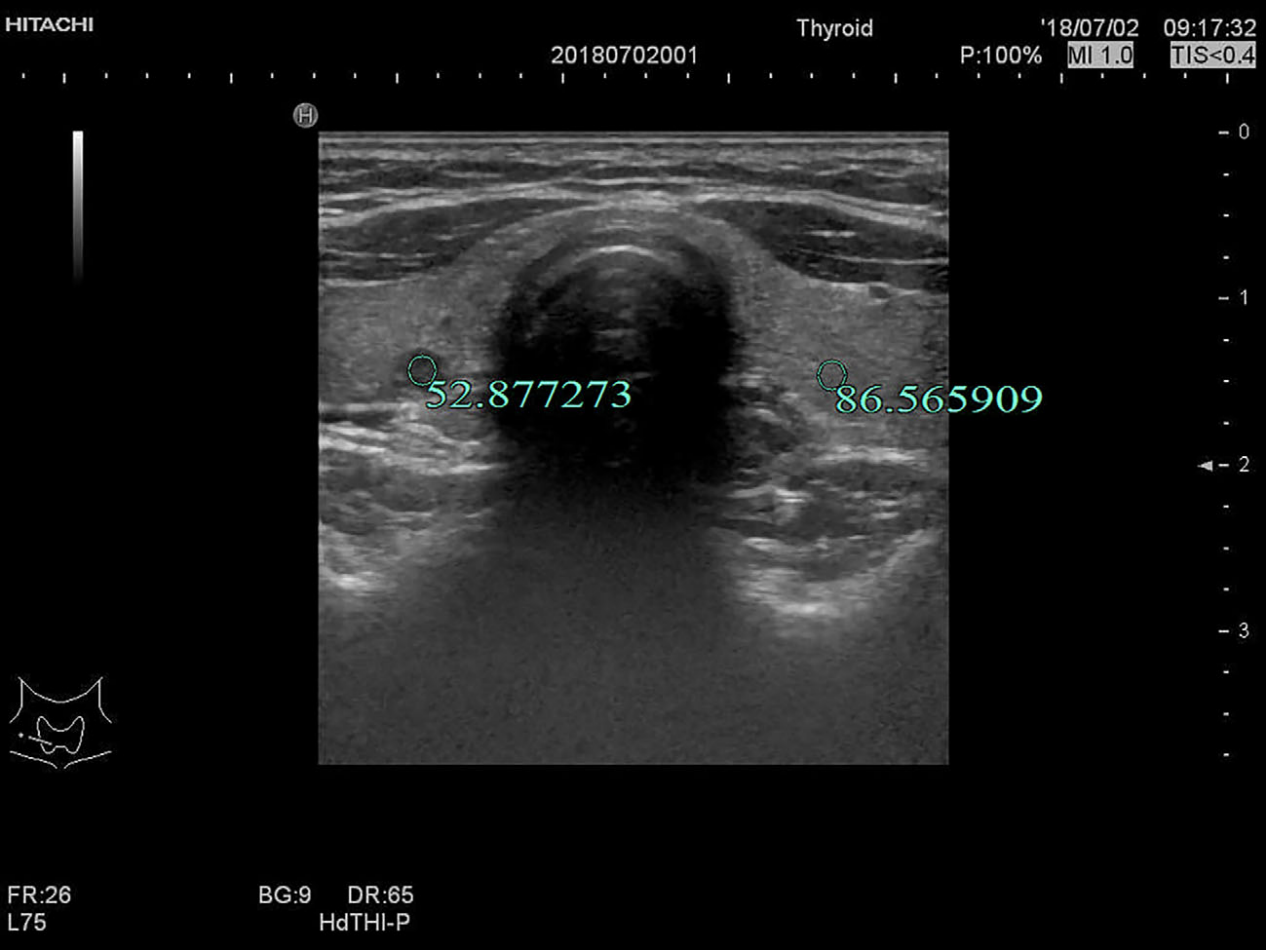
F, 27-years-old, PTMC in the right lobe of the thyroid, UGSR = 52.877273/86.565909 = 0.6108.

Two radiologists with more than 10 years of experience in two centers who were blinded to the pathological results assessed selected cases from the picture archiving and communication systems independently to measure thyroid nodules, the position of the region of interest (ROI), and the size of surrounding normal thyroid tissue. The assessment was performed using the gray histogram software of the RADinfo reading system (Zhejiang RAD Information Technology Co., Ltd., China). Ultrasound transverse/longitudinal section images were acquired to measure the gray-scale values ​​of the normal thyroid tissue and nodules. When the measured nodules had a uniform echo intensity, the ROI would be as large as possible (**Figure 2**). When the echo intensity of measured nodule was uneven and mainly composed of a certain echo intensity, ROI was selected in this echo intensity area, and the area was taken as large as possible (**Figure 3**). For cases with uneven echo intensity in which the ROI could not be determined as a specific type, the largest possible ROI was used for measurements (**Figure 4**). In the measurement of the three types of nodules, calcifications, cystic degeneration, and a surrounding hypoechoic halo were avoided. When the ROI around the nodule in the cross-section image was not sufficient, the same section on the opposite side was selected for measurement (**Figure 5**). The grayscale value of the normal thyroid tissue around the nodule was measured by selecting an ROI with the same size as that used for thyroid nodules and at the same gain level as the nodule. All measurements were performed twice, and the UGSR was calculated for each; the average of the two measurements was considered the UGSR of the nodule.

### Statistical Analysis

All statistical analyses were performed using SPSS 22.0 software (SPSS Inc., Chicago, IL, USA). The Mann-Whitney test was used for comparisons between the two groups. The receiver operating characteristic (ROC) curve of UGSR for PTMCs and MNGs was plotted with sensitivity as the ordinate and specificity as the abscisic coordinate. The area under the curve (AUC) was calculated. The optimal UGSR threshold was determined by comparing the sensitivity, specificity, Youden index, positive predictive value, negative predictive value, and accuracy.

## Results

1. Comparison of the Size of PTMCs and MNGs in Two Centers

The sizes of PTMCs and MNGs in center A were 6 (5–7) mm and 7 (5–9) mm, respectively (Z = -4.429, *P* = 0), whereas those in center B were 6 (4–8) mm and 8 (6–9) mm, respectively (Z = -5.348, *P* = 0), (**Table 1**). There is no statistical difference on the sizes of PTMCs between the two medical centers (Z = -0.844, *P* = 0.399), and there is also no statistical difference on the sizes of MNGs between the two medical centers (Z = -1.303, *P* = 0.193) (**Table 2**).

**Table 1:** Comparison of the size of PTMCs and MNGs between two medical centers.

	A		B	
	PTMCs(n = 881)	MNGs(n = 744)	PTMCs(n = 243)	MNGs(n = 251)
	6(5, 7)	7(5, 9)	6(4, 8)	8(6, 9)
Z	-4.429		-5.348	
*P*	0		0	

**Table 2:** Comparison of the size of PTMCs and MNGs between two medical centers.

	PTMCs	MNGs
A size(mm)	6(5, 7)	7(5, 9)
B size(mm)	6(4, 8)	8(6, 9)
Z	-0.844	-1.303
*P*	0.399	0.193

2. Comparison of the UGSRs Between PTMCs and MNGs in Two Centers

**Figure 6:**
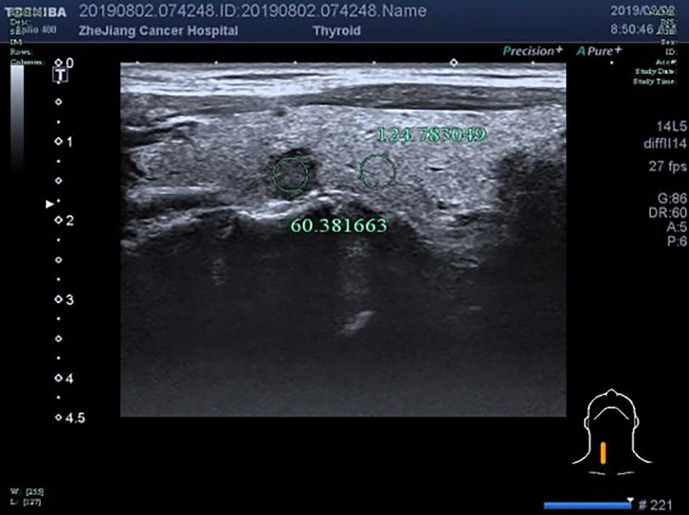
F, 48-years-old, PTMC in the right lobe of the thyroid, nodules with uniform echo intensity, UGSR = 60.381663/124.783049 = 0.4839.

The UGSRs measured from ultrasound images of 881 PTMCs from 785 cases (**Figure 5**) and 744 MNGs from 687 cases (**Figures 2** and **3**) in center A were 0.5537 (0.4699–0.6515) and 0.8708 (0.7616–1.0123), respectively (Z = - 27.691, *P* = 0). The UGSRs measured from the ultrasound images of 243 PTMCs from 203 cases (**Figure 6**) and 251 MNGs from 198 cases (**Figure 4**) in center B were 0.5517 (0.4698–0.6377) and 0.8539 (0.7366–0.9929), respectively (Z = -16.057, *P* = 0) (**Table 3**). There were no statistically significant differences in UGSR between PTMCs and MNGs in the two centers. The Z value and *P* value were -0.609 and -1.394, 0.543 and 0.163, respectively (**Table 4**).

**Table 3:** Comparison of UGSRs between PTMCs and MNGs in two medical centers.

	A		B	
	PTMCs(n = 881)	MNGs(n = 744)	PTMCs(n = 243)	MNGs(n = 251)
	0.5537 (0.4699, 0.6515)	0.8708 (0.7616, 1.0123)	0.5517 (0.4698, 0.6377)	0.8539 (0.7366, 0.9929)
Z	-27.691		-16.057	
*P*	0		0	

**Table 4:** Comparison of UGSRs between PTMCs and MNGs between two medical centers.

	PTMCs	MNGs
A	0.5537(0.4699, 0.6515)	0.8708(0.7616, 1.0123)
B	0.5517(0.4698, 0.6377)	0.8539(0.7366, 0.9929)
Z	-0.609	-1.394
*P*	0.543	0.163

3. Comparison of AUC values, optimal UGSR threshold, and diagnostic efficiency of the two medical centers

**Figure 7:**
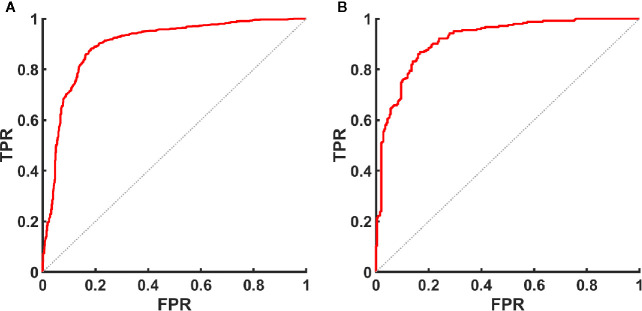
ROC curve of UGSR for identifying PTMCs and MNGs in the medical centers. **(A)** ROC curve of UGSR in center A. **(B)** ROC curve of UGSR in center B. TPR, true positive rate (sensitivity), FPR, false positive rate (1- specificity).

The AUC (**Figure 7**), optimal UGSR threshold, sensitivity, specificity, positive predictive value, negative predictive value, and accuracy of center A and center B were 0.898 vs. 0.918, 0.7214 vs. 0.6911, 0.881 vs. 0.868, 0.817 vs. 0.833, 0.851 vs. 0.834, 0.853 vs. 0.867, and 0.852 vs. 0.850, respectively (**Table 5**).

**Table 5:** Comparison of AUC, optimal UGSR threshold, and diagnostic efficiency of the two medical centers.

	AUC	Optimal UGSR threshold	Sensitivity	Specificity	Positive predictive value	Negative predictive value	Accuracy
A	0.898	0.7214	0.881	0.817	0.851	0.853	0.852
B	0.918	0.6911	0.868	0.833	0.834	0.867	0.850

## Discussion

Tumor morphology, microcalcifications, anteroposterior/transverse diameter ratio, and echo intensity are important ultrasonographic features for the identification of PTMCs and MNGs (1–3). Among these features, echo intensity can be affected by the subjective assessment of an ultrasound physician (4, 5). Quantification of echo intensity would improve the differential diagnosis of PTMCs and MNGs. In this study, we demonstrated that the UGSRs of PTMCs in the two medical centers were lower than those of MNGs. The AUC, optimal UGSR, and thresholds in the two medical centers were 0.898, 0.918, 0.7214, and 0.6911, respectively. Parameters such as sensitivity, specificity, positive predictive value, negative predictive value, and accuracy showed similar values, ranging from 0.81 to 0.89. This indicates that the UGSRs were highly consistent between the two medical centers, and the diagnostic efficiency was considerably higher than that of the 3–5 level method in the literature (9–15). In addition, the present results showed no statistically significant differences in the size of PTMCs and MNGs between the two medical centers, whereas the size of PTMCs in the two centers was smaller than that of MNGs. This may be attributed to the fact that the small MNGs that were excluded were mostly cystic glial nodules and did not meet the inclusion criteria.

There are currently only four studies addressing UGSR quantification of thyroid nodules. In 2015, Giorgio et al. (7) measured the grayscale values ​​of nodules, peripheral thyroid, and neck band muscles, and calculated the nodule/peripheral thyroid, nodule/muscle, and peripheral thyroid/muscle USGRs. The authors reported that the malignant nodule/peripheral thyroid USGR is significantly lower than that of benign nodules, and the observers reached an agreement (k = 0.74). When the ratio is <0.46, the sensitivity and specificity for predicting malignant nodules are 56.7% and 72%, respectively, and the nodule/muscle UGSR is not as valuable as the nodule/peripheral thyroid UGSR for predicting malignant nodules. Although Giorgio et al. pioneered the use of UGSR to quantify the echo intensity of nodules, their study has many limitations. First, the data were derived from a single medical center and obtained using the same ultrasound scanner. Whether the conclusions can be applied to other medical centers or ultrasound scanners needs further verification. Second, there are no studies classifying thyroid nodules according to size. The echo intensity of nodules can vary according to size (9, 15). A study including nodules of different sizes decreases the practical value of the UGSR. Third, the sample size of malignant nodules was small, and nodules were not classified according to pathological subtype. Fourth, all nodules showed signs of malignancy on ultrasound, such as unclear borders, microcalcifications, and hypoechoic areas, and they were diagnosed by FNAC; therefore, most nodules showing benign signs were not included in the UGSR study. In 2018, we used UGSR for the differential diagnosis of PTMCs and MNGs in a single medical center. The results showed that the AUC, optimal UGSR threshold, sensitivity, and specificity were 0.895, 0.72, 87.0, and 80.4 (5), respectively. The same parameters were used in this study, and the results were highly consistent between the two medical centers. In the same year, we performed a comparative study of anechoic MNGs and very hypoechoic PTMCs. The results showed that the UGSR of anechoic MNGs was lower, the optimal threshold was 0.26, and the sensitivity and specificity for predicting anechoic MNGs were 94.3% and 99.0%, respectively (6). In 2019, Chen et al. (8) divided papillary carcinomas and nodular goiters into three groups according to size as follows: 0.3–1.0, 1.0–1.5, and 1.5–2.0 cm. The results showed that the AUC, optimal UGSR threshold, sensitivity, and specificity vary with the size of the tumor. An increase in tumor size is associated with a decrease in the AUC and sensitivity and an increase in the optimal UGSR threshold and specificity. The AUC and optimal UGSR threshold of the 0.3–1.0 cm group were highly consistent with that of the present study at 0.919 and 0.692, respectively. Although the specificity (0.724) was lower than that of the present study, the sensitivity (0.975) was higher than that of this study, and the diagnostic performance was similar, which largely supports our results. The limitation in the data from our previous studies (5, 6) and those from the study by Chen et al. (8) is that they were obtained in a single medical center, and the data reported by Chen et al. were obtained using two different scanners from the same ultrasound machine manufacturer.

Compared with the studies of Giorgio et al. (7) and Chen et al. (8), the present study has the following three advantages. First, the study included two medical centers, as well as different ultrasound scanners, operators, and cases between the two medical centers, which increases the clinical value of the results. Second, the sample size was large, including 1124 PTMCs and 995 MNGs. Third, all nodules were confirmed by surgery and pathology. Most of the MNGs in this study were confirmed by surgery due to combined with malignant nodules or larger benign nodules, without screening, thus the UGSR was more representative of benign nodules. The present results indicated that UGSR was valuable for the differential diagnosis of PTMCs and MNGs, and was stabilized.

The present study had several limitations. First, for normal thyroid tissue with uneven echo intensity or nodules with uneven echo intensity related to technical factors, the ROI was selected and measured differently. In this study, the data of the two centers were measured twice by a senior imaging physician in each center, and the mean values were calculated, which could reduce the difference in the ROI to a large extent. In addition, it is also an important purpose of our study to arouse the operator’s awareness of standardized scanning. Second, there are no ultrasound signs that completely distinguish benign from malignant thyroid nodules (16, 17). The two types of nodules are differentiated using a combination of signs. However, the aim of this study was to provide information on UGSR for clinicians and imaging physicians. The combination of UGSR with other ultrasound signs for identifying benign and malignant thyroid nodules will be the direction of our future research. Third, the diameters of PTMCs and MNGs in our study were 0.4–1.0 cm, and the maximum diagnostic efficacy were achieved when the UGSR were 0.7214 and 0.6911, respectively. The effect of diameters <0.4 or >1.0 cm on UGSR remains to be determined. Finally, this study was a retrospective analysis of two centers, and additional prospective studies in medical centers should be performed to verify the value and stability of UGSR.

In conclusion, UGSR was of great value for the differential diagnosis of PTMCs and MNGs by quantifying the echo intensity of the two lesions. The diagnostic performance of the two medical centers were consistent and the method is simple to apply, providing an important reference for improving TIRADS.

## Data Availability Statement

The original contributions presented in the study are included in the article/supplementary material; further inquiries can be directed to the corresponding author.

## Ethics Statement

The studies involving human participants were reviewed and approved by the ethics committees, Affiliated Hangzhou First People’s Hospital, Zhejiang University School of Medicine and The Cancer Hospital of the University of Chinese Academy of Sciences (Zhejiang Cancer Hospital). Written informed consent was obtained from the individual(s) for the publication of any potentially identifiable images or data included in this article.

## Author Contributions

DX and ZH conceived and designed of the study. NF, YL, ML, JY, QZ, and ZL acquired the data. PW and YL analyzed and/or interpreted of the data. ZH and YL drafted the manuscript. ZH, YL, and DX revised the manuscript critically for important intellectual content. ZH, NF, and YL contributed equally to this article. All authors contributed to the article and approved the submitted version.

## Funding

This work was funded by the National Natural Science Foundation of China (81871370, 82071946), Key Provincial Natural Science Foundation of Zhejiang (LSD19H180001), Key Project of Scientific and Technological Innovation in Hangzhou (20131813A08), Medical Science Research Program of Zhejiang Province (2020RC091, 2021RC024, 2017ZD009), and Institute of Basic Medicine and Cancer (IBMC), Chinese Academy of Sciences, Key Laboratory of Head & Neck Cancer Translational Research of Zhejiang Province, Zhejiang Provincial Research Center for Cancer Intelligent Diagnosis and Molecular Technology, Hangzhou, Zhejiang 310022, China.

## Conflict of Interest

The authors declare that the research was conducted in the absence of any commercial or financial relationships that could be construed as a potential conflict of interest.

## Abbreviations

PTMCs, microcarcinomas; MNGs, micronodular goiters; UGSR, ultrasound gray-scale ratio; ROC, receiver operating characteristic; AUC, area under the curve; center A, Affiliated Hangzhou First People’s Hospital, Zhejiang University School of Medicine; center B, The Cancer Hospital of the University of Chinese Academy of Sciences (Zhejiang Cancer Hospital); TIRADS, Thyroid Imaging Reporting and Data System; ACR, American College of Radiology; PACS, picture archiving and communication systems.

## References

[1] Shin JH Baek JH Chung J Ha EJ Kim JH Lee YH Ultrasonography diagnosis and imaging-based management of thyroid nodules: Revised Korean Society of Thyroid Radiology Consensus Statement and Recommendations. Korean J Radiol (2016) 17(3):370–95. 10.3348/kjr.2016.17.3.370 PMC4842857 27134526

[2] Tessler FN Middleton WD Grant EG Hoang JK Berland LL Teefey SA ACR thyroid imaging, reporting and data system (TI-RADS): White paper of the ACR TI-RADS Committee. J Am Coll Radiol (2017) 14(5):587–95. 10.1016/j.jacr.2017.01.046 28372962

[3] Russ G Bonnema SJ Erdogan MF Durante C Ngu R Leenhardt L European Thyroid Association Guidelines for Ultrasound Malignancy Risk Stratification of Thyroid Nodules in Adults: The EU-TIRADS. Eur Thyroid J (2017) 6(5):225–37. 10.1159/000478927 PMC5652895 29167761

[4] Liu H Ma A-L Zhou Y-S Yang D-H Ruan J-L Liu X-D Variability in the interpretation of grey-scale ultrasound features in assessing thyroid nodules: A systematic review and meta-analysis. Eur J Radiol (2020) 129:109050. 10.1016/j.ejrad.2020.109050 32447147

[5] Han Z-J Lei Z-K Li M-K Luo D-C Ding J-W Differential diagnosis value of the ultrasound gray scale ratio for papillary thyroid microcarcinomas and micronodular goiters. Quant Imaging Med Surg (2018) 8(5):507–13. 10.21037/qims.2018.06.04 PMC6037957 30050785

[6] Lei Z-K Li M-K Luo D-C Han Z-J The clinical significance of ultrasound grayscale ratio (USGR) in differentiating markedly hypoechoic and anechoic minimal thyroid nodules. J Cancer Res Ther (2018) 14(7):1567–71. 10.4103/jcrt.JCRT_1031_17 30589040

[7] Grani G D’Alessandri M Carbotta G Nesca A Del Sordo M Alessandrini S Grey-Scale Analysis Improves the Ultrasonographic Evaluation of Thyroid Nodules. Med (Baltimore) (2015) 94(27):e1129. 10.1097/MD.0000000000001129 PMC4504637 26166117

[8] Chen X-Y Gao M Hu L-F Zhu J-L Zhang S Wei X The diagnostic value of the ultrasound gray scale ratio for different sizes of thyroid nodules. Cancer Med (2019) 8(18):7644–9. 10.1002/cam4.2653 PMC6912051 31691509

[9] Kim GR Kim MH Moon HJ Chung WY Kwak JY Kim EK Sonographic characteristics suggesting papillary thyroid carcinoma according to nodule size. Ann Surg Oncol (2013) 20(3):906–13. 10.1245/s10434-012-2830-4 23266584

[10] Moon W-J Jung SL Lee JH Na DG Baek JH Lee YH Benign and malignant thyroid nodules: US differentiation–multicenter retrospective study. Radiology (2008) 247(3):762–70. 10.1148/radiol.2473070944 18403624

[11] Cappelli C Castellano M Pirola I Gandossi E De Martino E Cumetti D Thyroid nodule shape suggests malignancy. Eur J Endocrinol (2006) 155(1):27–31. 10.1530/eje.1.02177 16793946

[12] Sharma A Gabriel H Nemcek AA Nayar R Du H Nikolaidis P Subcentimeter thyroid nodules: utility of sonographic characterization and ultrasound-guided needle biopsy. AJR Am J Roentgenol (2011) 197(6):W1123–1128. 10.2214/AJR.10.5684 22109329

[13] Ren J Liu B Zhang L-L Li H-Y Zhang F Li S A taller-than-wide shape is a good predictor of papillary thyroid carcinoma in small solid nodules. J Ultrasound Med (2015) 34(1):19–26. 10.7863/ultra.34.1.19 25542935

[14] Hong YJ Son EJ Kim EK Kwak JY Hong SW Chang HS Positive predictive values of sonographic features of solid thyroid nodule. Clin Imaging (2010) 34(2):127–33. 10.1016/j.clinimag.2008.10.034 20189077

[15] Ha SM Kim JK Baek JH Detection of malignancy among suspicious thyroid nodules <1 cm on ultrasound with various thyroid image reporting and data systems. Thyroid (2017) 27(10):1307–15. 10.1089/thy.2017.0034 28791920

[16] Papini E Guglielmi R Bianchini A Crescenzi A Taccogna S Nardi F Risk of malignancy in nonpalpable thyroid nodules: predictive value of ultrasound and color-Doppler features. J Clin Endocrinol Metab (2002) 87(5):1941–6. 10.1210/jcem.87.5.8504 11994321

[17] Ma J-J Ding H Xu B-H Xu C Song L-J Huang B-J Diagnostic performances of various gray-scale, color Doppler, and contrast-enhanced ultrasonography findings in predicting malignant thyroid nodules. Thyroid (2014) 24(2):355–63. 10.1089/thy.2013.0150 23978252

